# Re-evaluating physiological indicators for all-cause mortality

**DOI:** 10.1016/S2666-7568(21)00228-2

**Published:** 2021-09-20

**Authors:** Sean Clouston, Graciela Muniz Terrera

**Affiliations:** Department of Family, Population, and Preventive Medicine, Renaissance School of Medicine at Stony Brook University, Stony Brook, NY 11794-8338, USA; Clinical Brain Sciences, Edinburgh Dementia Prevention, The University of Edinburgh, Edinburgh, UK

Geriatric research has consistently focused on the identification of appropriate benchmarks for clinical decision making and the improved detection of individuals who might be at increased risk of poor health. Over decades, researchers have relied on a small number of analytical tools, including linear models (eg, linear and logistic regression or survival modelling) to accumulate a wealth of information about appropriate biomarkers by assessing the associations between these markers and risk of death. This research has shown that patients who have systolic blood pressure exceeding 130 mm Hg,^1^ or who have very low HDL cholesterol concentrations (≤30 mg/dL) have an increased risk of mortality.^2^ Yet many researchers insist that such cutoffs should be viewed with scepticism, because the fulfilment of modelling assumptions are not always fully evaluated or described.^3^

Clinical decision making, research protocols, and guidelines for healthy behaviours often rely on established cutoffs.^4^ As a result, knowledge and strategies for effective care plans and therapeutic treatment of older adults could change when agreed upon cutoffs cannot be replicated or they change. At the same time, it is increasingly clear that average scores on several clinical examinations have shifted over time, as the impact of infectious disease, famine, and trauma on health outcomes and mortality risk across the life course has decreased.^5^ Concurrently, there is increasing interest in early interventions based on cutoffs used to define healthy standards, as early intervention has the potential to improve long-term outcomes for age-related diseases including Alzheimer’s disease and related dementias. Despite these concerns, cutoffs are rarely re-examined using novel populations, resulting in diagnostic criteria that might rely on outdated cutoffs.

In *The Lancet Healthy Longevity,* Vy Kim Nguyen and colleagues^6^ challenged existing clinical thresholds for a large set of physiological indicators and their association with all-cause mortality. They used a large normative database (n=47 266 adults) to investigate how well commonly assumed linear models characterise the association between 27 physiological indicators and mortality, when compared with different non-linear models, using a data-driven approach and Cox proportional hazards modelling. 25 (93%) of 27 indicators showed non-linear associations, with substantial increases in mortality risk. Their work highlights the importance of characterising the shape of the association of physiological indicators with key health outcomes. Concerns about the impact of non-linear associations in health sciences have been previously discussed in the methodological literature,^7^ hence their work is timely. Their findings replicated several cutoffs commonly used in the medical literature (eg, diastolic blood pressure and creatinine, among others) but also challenged some cutoffs that might be outdated (eg, high or low body-mass index and high or low waist circumference, among others). They identified novel cutoffs that could indicate novel syndromes that, although sometimes mild, might be worth further evaluation (eg, HDL cholesterol >91 mg/dL for women and >68 mg/dL for men). The finding that high glomerular filtration rate (>90 mL/min per 1·73 m^2^) was associated with heightened mortality risk is concerning because these risks are not well understood and could include medical, psychological, and behavioural factors.^8^ As mortality risk appeared higher in participants with high glomerular filtration rate than in those with normal or low glomerular filtration rate indicative of chronic kidney disease, high glomerular filtration rate is worth further investigation.

It is unlikely that this Article will conclude discussions about the mortality risk associated with any of these physiological indicators. As Bartlett and colleagues^9^ clarified when deriving cutoff points for Alzheimer’s disease biomarkers, the selection of cutoff points should be made when specifically considering the use of the physiological indicator since deriving a unique cutoff point for a given physiological indicator is a formidable task. The authors provide an important service by highlighting several limitations of the current process used to derive clinical thresholds, which are important to consider and that could offer opportunities for future research. However, the work by Nguyen and colleagues adds a data driven methodology to the supply of analytical approaches that could push these efforts forwards and improve our understanding of the complex associations between biomarkers and health outcomes. We praise the authors for adopting data and code sharing practices that will undoubtedly facilitate the replication of their work. The replication of this work in other datasets will permit the evaluation of generalisability of their findings to other populations.

Researchers are increasingly recognising that the usual methods for identifying diseases often rely on old and out-of-date data and that the processes used in determining disease status could themselves be outdated. Perhaps the most promising element of this study is that it portends a time when appropriate studies continually update risk predictions and help us to create a process for updating our assessment of risk and, therefore, inform us of the need for intervention.
